# Bio-Tribocorrosion of Titanium Dental Implants in the Oral Environment: A Narrative Review

**DOI:** 10.7759/cureus.103188

**Published:** 2026-02-08

**Authors:** Aarcha Hariprasad, Shankar S Menon, Arun Kurumathur Vasudevan, Maya Rajan Peter, Biju Balakrishnan, Reshma Suresh

**Affiliations:** 1 Periodontics, Amrita School of Dentistry, Amrita Vishwa Vidyapeetham, Kochi, IND

**Keywords:** bio-tribocorrosion, dental implants, oral biofilm, peri-implantitis, titanium, titanium particles, tribocorrosion

## Abstract

Titanium and its alloys are widely used in dental implantology because of their favorable mechanical properties, corrosion resistance, and biocompatibility. However, long-term clinical success of dental implants is increasingly challenged by material degradation processes occurring in the complex oral environment. Tribocorrosion, a synergistic phenomenon involving mechanical wear and electrochemical corrosion, has emerged as a critical mechanism contributing to implant surface deterioration. When biological factors such as oral biofilms, saliva, inflammatory mediators, and fluctuating pH are incorporated into this interaction, the process is termed bio-tribocorrosion. This narrative review provides a comprehensive overview of the fundamental principles of tribocorrosion and extends them to the context of bio-tribocorrosion affecting titanium dental implants. The mechanisms underlying the synergism between mechanical loading and chemical or electrochemical degradation are discussed, along with factors influencing bio-tribocorrosion in the oral cavity, including implant material composition, surface characteristics, biofilm formation, fluoride exposure, salivary components, mastication-induced micromovements, and galvanic interactions. The biological consequences of titanium ion and particle release are critically examined, with emphasis on cytotoxicity, inflammatory responses, immune modulation, hypersensitivity reactions, peri-implant tissue breakdown, and bone loss. Evidence linking titanium wear debris to peri-implantitis and implant failure is reviewed, and current in vitro tribocorrosion testing methodologies and their limitations are summarized. Finally, emerging strategies aimed at mitigating bio-tribocorrosion, such as alloy development, surface modifications, protective coatings, and alternative biomaterials, are discussed. Understanding bio-tribocorrosion is essential for improving implant longevity, minimizing biological complications, and guiding future innovations in dental implant materials and design.

## Introduction and background

Dental implants have become a cornerstone of contemporary oral rehabilitation, offering predictable functional and aesthetic outcomes for partially and completely edentulous patients. Among the various biomaterials used for implant fabrication, commercially pure titanium (CpTi) and titanium alloys, particularly Ti-6Al-4V, have remained the materials of choice due to their favorable mechanical strength, corrosion resistance, and excellent biocompatibility mediated through osseointegration [1]. The long-term success of dental implants, however, depends not only on surgical precision and prosthetic design but also on the stability of the implant material within the biologically active and mechanically dynamic oral environment [2].

Despite high reported survival rates, implant-related biological complications such as peri-implant mucositis and peri-implantitis continue to pose significant clinical challenges. Increasing evidence suggests that material degradation phenomena at the implant surface may play an underappreciated role in the initiation and progression of peri-implant tissue breakdown [3]. Among these degradation mechanisms, tribocorrosion, defined as the synergistic interaction between mechanical wear and electrochemical corrosion, has emerged as a critical factor influencing the integrity and longevity of metallic biomaterials [4].

Tribocorrosion represents a departure from the traditional isolated evaluation of wear and corrosion processes. While tribology focuses on friction, wear, and lubrication, and corrosion addresses electrochemical material degradation, tribocorrosion integrates both domains to describe the accelerated material loss that occurs when mechanical and chemical processes act simultaneously [5]. In metallic systems protected by passive oxide films, such as titanium, this synergism becomes particularly relevant. Mechanical disruption of the oxide layer exposes the underlying metal to the surrounding environment, triggering localized corrosion, followed by repassivation, repeated cyclically under dynamic loading conditions [6]. The cumulative effect of this cycle results in material degradation that is often substantially greater than the sum of wear or corrosion acting independently [7].

The oral cavity presents a uniquely complex environment in which tribocorrosion processes are amplified. Dental implants are subjected to cyclic occlusal forces during mastication, parafunctional habits, and prosthetic micromovements, while simultaneously being exposed to saliva, fluctuating pH, dietary acids, fluoride-containing oral hygiene products, and a dense and metabolically active microbial biofilm [8]. These interacting mechanical, chemical, and biological factors create conditions conducive to accelerated degradation of titanium implant surfaces [9].

When biological variables such as oral biofilms, inflammatory mediators, microbial metabolites, and host immune responses are incorporated into the tribocorrosion framework, the process is more accurately described as bio-tribocorrosion [10]. Bio-tribocorrosion acknowledges that living systems are not passive environments; rather, they actively modify electrochemical conditions through enzymatic activity, pH modulation, and inflammatory responses, thereby influencing material behavior in ways not replicated in purely physicochemical testing models [11].

Titanium owes its corrosion resistance primarily to the formation of a stable, nanometer-thick titanium dioxide (TiO₂) passive film on its surface. This oxide layer acts as a barrier to ion diffusion and electrochemical reactions, thereby limiting metal dissolution [12]. However, under conditions of mechanical loading, such as fretting at the implant-abutment interface, micromovements at the bone-implant interface, or surface abrasion during implant placement and maintenance, the passive film can be mechanically disrupted [13]. Repeated depassivation and repassivation cycles not only accelerate material loss but also facilitate the release of titanium ions and particles into surrounding tissues [14].

Emerging evidence indicates that titanium wear debris is not biologically inert. Titanium particles and ions released through bio-tribocorrosion have been detected in peri-implant soft tissues, bone, regional lymph nodes, and even distant organs, as reported predominantly in preclinical and experimental contexts, with limited and indirect human evidence [15]. These particles vary widely in size, ranging from micrometer-scale debris to nanoparticles, each exhibiting distinct physicochemical and biological properties [16]. Smaller particles, particularly nanoparticles, possess a high surface area-to-volume ratio, rendering them highly reactive and capable of interacting with cellular and molecular pathways involved in inflammation and bone metabolism. Much of the nanoparticle bioactivity evidence, though, derives from in vitro dose-response models, often using concentrations exceeding typical clinical exposure [17].

At the cellular level, titanium particles have been shown to induce cytotoxicity, oxidative stress, and pro-inflammatory cytokine release in epithelial cells, fibroblasts, macrophages, and osteoblasts [18]. Macrophage activation by titanium debris can lead to the release of inflammatory mediators such as interleukin-1β, tumor necrosis factor-α, and receptor activator of nuclear factor kappa-B ligand (RANKL), thereby promoting osteoclastogenesis and peri-implant bone resorption [19]. These biological responses provide a mechanistic plausibility between material degradation and peri-implant disease progression [20].

The presence of oral biofilms further complicates the bio-tribocorrosion process. Early and late colonizing microorganisms produce organic acids, lipopolysaccharides, and extracellular polymeric substances that alter local pH and electrochemical conditions at the implant surface [21]. Biofilm accumulation at microgaps and roughened surfaces creates differential oxygen concentrations, promoting localized corrosion phenomena such as pitting and crevice corrosion [22]. Moreover, microbial metabolites may interfere with the repassivation kinetics of titanium, thereby prolonging exposure of the metal surface to corrosive conditions following mechanical damage [23].

Fluoride exposure represents another critical modifier of bio-tribocorrosion in the oral cavity. While fluoride plays a key role in caries prevention, fluoride ions, particularly under acidic conditions, can destabilize the TiO₂ layer by forming soluble titanium-fluoride complexes [24]. Clinical scenarios such as the use of acidic fluoridated gels, mouth rinses, and prophylactic agents may therefore increase the susceptibility of titanium implants to corrosion, especially when combined with mechanical wear [25].

In addition to chemical and biological influences, implant material composition and surface characteristics significantly affect bio-tribocorrosion behavior. Titanium alloys containing aluminum and vanadium, such as Ti-6Al-4V, exhibit different corrosion and wear profiles compared to CpTi due to variations in oxide composition, microstructure, and mechanical hardness [26]. Newer alloys and surface-modified implants have been developed to enhance corrosion resistance and reduce wear; however, their long-term performance under bio-tribocorrosive conditions remains an area of active investigation [27].

Despite increasing recognition of bio-tribocorrosion as a clinically relevant phenomenon, its contribution to implant failure remains underappreciated in routine dental practice. Most clinical assessments of implant success focus on osseointegration, prosthetic stability, and microbial control, with limited consideration of material degradation at the implant surface [28]. Furthermore, the majority of available evidence is derived from in vitro studies employing simplified testing conditions that may not fully replicate the complexity of the oral environment [29]. It is important to note that the current understanding of tribocorrosion and bio-tribocorrosion in implant dentistry is derived from a heterogeneous body of evidence, including in vitro mechanistic studies, animal models, implant retrieval analyses, and a limited number of associative clinical investigations. Consequently, while mechanistic plausibility and biological relevance are well supported, direct causal relationships with peri-implant disease progression or systemic effects in humans have not been established [28,29].

A comprehensive understanding of bio-tribocorrosion is therefore essential for improving implant design, refining surface modification strategies, and developing evidence-based clinical protocols aimed at minimizing material degradation and its biological consequences. By integrating insights from tribology, corrosion science, microbiology, immunology, and clinical implantology, bio-tribocorrosion provides a unifying framework to explain how mechanical, chemical, and biological factors converge to influence the long-term performance of dental implants [30].

Accordingly, the present review aims to synthesize current knowledge on tribocorrosion and bio-tribocorrosion as it relates to titanium dental implants. Emphasis is placed on the underlying mechanisms, influencing factors within the oral environment, biological effects of titanium degradation products, and their potential role in peri-implant disease. Additionally, existing methodologies for evaluating bio-tribocorrosion and emerging strategies to mitigate implant degradation are discussed to provide a comprehensive perspective on this evolving field.

## Review

Methodology

This article was conducted as a narrative scientific review aimed at synthesizing current evidence on tribocorrosion and bio-tribocorrosion of titanium dental implants, with particular emphasis on underlying mechanisms, biological consequences, and clinical relevance. A narrative review methodology was selected to allow integration of heterogeneous evidence derived from materials science, tribology, electrochemistry, microbiology, and clinical implant dentistry, all of which are domains that are rarely amenable to quantitative pooling due to differences in experimental models, outcomes, and study designs [1].

Literature Search Strategy

A structured electronic literature search was performed using PubMed/MEDLINE, Scopus, and Web of Science. The search covered publications from database inception through January 2026. Search strategies combined controlled vocabulary and free-text terms using Boolean operators, including: (“tribocorrosion” OR “bio-tribocorrosion”) AND (“titanium” OR “commercially pure titanium” OR “Ti-6Al-4V”) AND (“dental implants” OR “implant-abutment interface” OR “peri-implantitis”), along with related terms such as wear, corrosion, fretting, oral biofilm, fluoride, titanium particles, and peri-implant disease. Reference lists of selected articles were manually screened to identify additional foundational and frequently cited studies relevant to tribocorrosion in implant dentistry [2].

Study Selection and Flow

**Figure 1:**
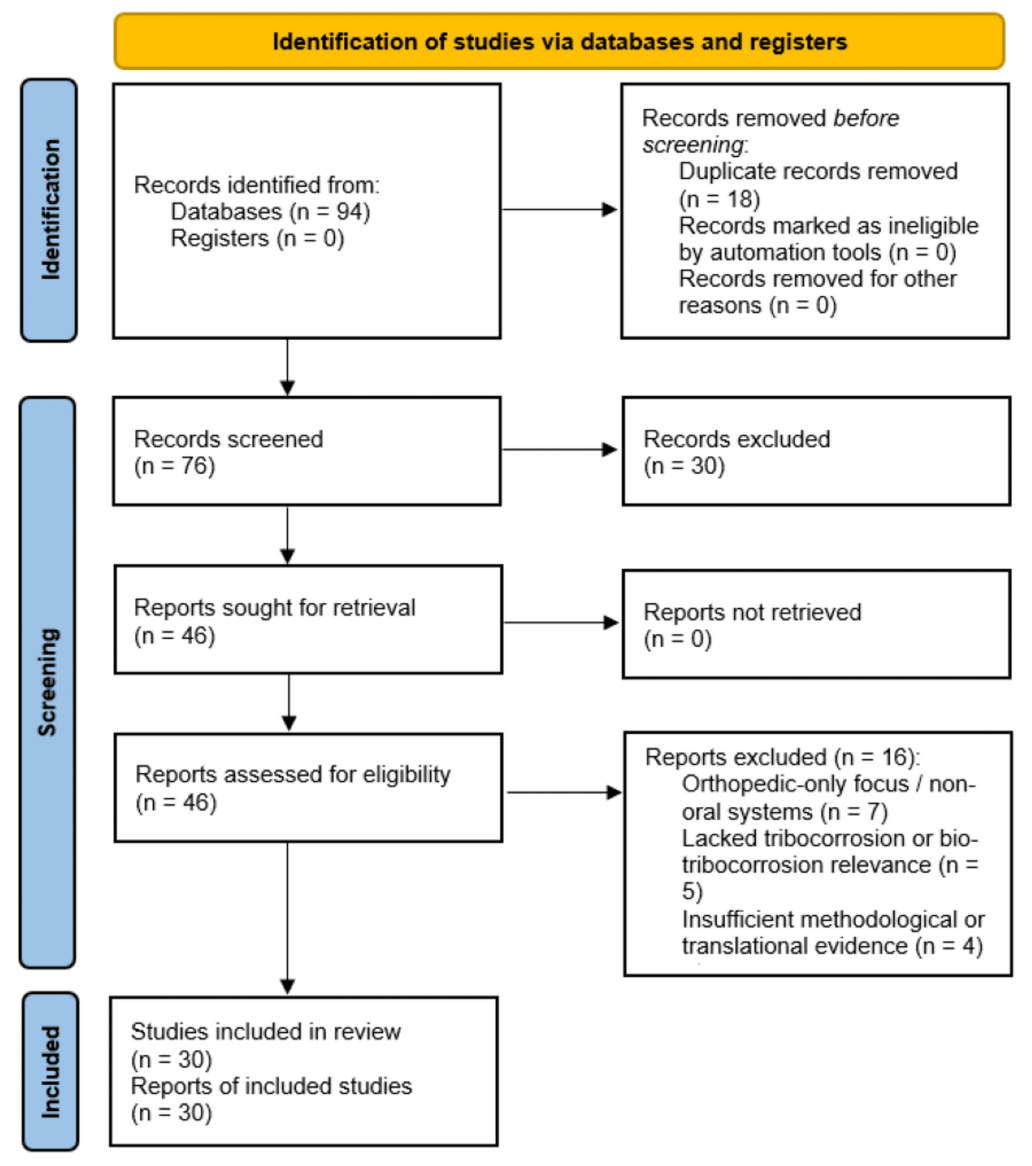
PRISMA 2020 flow diagram illustrating study identification, screening, eligibility, and inclusion Credits: Dr. Shankar S Menon PRISMA: Preferred Reporting Items for Systematic Reviews and Meta-Analyses

The electronic search initially retrieved 94 records. After removal of 18 duplicate entries, 76 unique records were screened based on title and abstract. Of these, 46 articles were excluded for lack of relevance to dental implants, absence of tribocorrosion-related mechanisms, or exclusive focus on non-oral orthopedic systems without translational relevance. Thirty full-text articles met the inclusion criteria and were incorporated into the narrative synthesis (Figure 1).

Inclusion and Exclusion Criteria

Studies were eligible for inclusion if they investigated tribological wear, electrochemical corrosion, or their synergistic interaction involving titanium or titanium-based alloys used in dental implants under conditions relevant to the oral environment. Experimental studies examining biological responses to titanium ions or particles, including inflammatory and bone-related pathways, were included to capture the biological dimension of bio-tribocorrosion. Clinical, retrieval, and observational studies reporting titanium degradation products in peri-implant tissues were also included. Studies focusing exclusively on orthopedic implants, non-peer-reviewed literature, conference abstracts, and publications lacking methodological relevance to oral implantology were excluded unless the underlying mechanisms were directly translatable [3].

Screening, Data Extraction, and Study Quality Considerations

Title/abstract screening and full-text assessment were conducted independently by two reviewers, with disagreements resolved through discussion and consensus. Data extraction was qualitative and focused on study type, experimental or clinical model, material composition, surface characteristics, mechanical loading conditions, electrochemical environment, biological context, and principal findings. Consistent with the narrative review design, no formal risk-of-bias assessment or standardized quality scoring was performed. Instead, study quality and relevance were considered descriptively based on methodological rigor, relevance to oral implant conditions, evidence level (in vitro, animal, retrieval, or clinical), and translational applicability. This approach was chosen to avoid inappropriate application of systematic review appraisal tools to fundamentally heterogeneous evidence [3,4].

Evidence Synthesis

No quantitative synthesis or meta-analysis was performed, as substantial heterogeneity existed across study designs, testing protocols, outcome measures, and biological models. Accordingly, no pooled estimates, P-values, confidence intervals, or statistical analyses were generated, and a statistician review was not required. Findings were synthesized thematically and qualitatively, organized into domains addressing tribocorrosion mechanisms, oral environmental modifiers, biological responses to titanium degradation products, clinical associations, and mitigation strategies. To improve interpretability and avoid causal overinterpretation, evidence is explicitly distinguished throughout the manuscript as mechanistic (in vitro), preclinical (animal), retrieval-based, or associative clinical, and an evidence-level summary table has been provided [3,4].

Results

The literature reviewed across in vitro, animal, retrieval, and limited clinical studies consistently demonstrated that tribocorrosion represents a significant degradation mechanism affecting titanium dental implants when mechanical wear and electrochemical corrosion act synergistically under oral conditions. Across in vitro, in vivo, and retrieval studies, material loss observed under combined mechanical and corrosive challenges was substantially greater than that produced by wear or corrosion alone, confirming the non-linear and synergistic nature of tribocorrosion phenomena [6]. This effect was particularly evident in systems involving repeated mechanical loading, such as fretting at the implant-abutment interface and micromovements at the bone-implant interface.

Experimental tribocorrosion studies revealed that disruption of the protective titanium oxide layer plays a central role in initiating degradation. Mechanical actions such as sliding, abrasion, or fretting were shown to locally remove or thin the passive TiO₂ film, exposing the underlying titanium substrate to the surrounding electrolyte and resulting in a transient increase in corrosion current density [7]. Although repassivation of the oxide layer occurred rapidly, repeated depassivation-repassivation cycles under cyclic loading led to cumulative surface damage and progressive material loss. Electrochemical measurements consistently demonstrated fluctuations in open circuit potential and corrosion current during mechanical wear, highlighting the dynamic interaction between mechanical and electrochemical processes [8].

Surface characteristics were found to significantly influence tribocorrosion behavior. Roughened implant surfaces, while beneficial for osseointegration, exhibited higher susceptibility to wear-induced oxide disruption due to increased surface area and stress concentration at asperities [9]. Conversely, smoother surfaces demonstrated lower wear rates but were not immune to tribocorrosion, particularly under high-load or fretting conditions. Studies comparing commercially pure titanium with titanium alloys reported differences in tribocorrosion resistance attributable to variations in microstructure, hardness, and oxide composition, with Ti-6Al-4V often exhibiting higher wear resistance but more complex corrosion behavior [10].

The oral environment emerged as a critical modifier of tribocorrosion processes. Variations in pH, particularly acidic conditions associated with dietary intake, microbial metabolism, or inflammatory states, were shown to increase corrosion rates and impair repassivation of titanium surfaces [11]. The presence of fluoride ions further exacerbated material degradation, especially under acidic conditions, through the formation of soluble titanium-fluoride complexes that destabilized the oxide layer [12]. Tribocorrosion experiments incorporating fluoride-containing electrolytes consistently reported increased material loss and higher corrosion currents compared to fluoride-free environments.

Biological factors were identified as key contributors to the transition from tribocorrosion to bio-tribocorrosion. Studies incorporating oral biofilms demonstrated that microbial adhesion and biofilm maturation altered local electrochemical conditions at the implant surface, creating microenvironments characterized by reduced pH, differential oxygen concentrations, and accumulation of corrosive metabolic by-products [13]. These conditions promoted localized corrosion phenomena, including pitting and crevice corrosion, particularly in protected regions such as the implant-abutment microgap [14]. Biofilm-covered surfaces also exhibited delayed repassivation following mechanical damage, prolonging exposure of the metal surface to corrosive conditions.

A consistent finding across biological studies was the release of titanium ions and particles as a consequence of bio-tribocorrosion. Analytical techniques such as inductively coupled plasma mass spectrometry and electron microscopy confirmed the presence of titanium degradation products in peri-implant soft tissues, bone, and biofilms [15]. Retrieved implants from certain failed clinical cases exhibited surface wear, corrosion features, and accumulation of titanium debris in adjacent tissues, supporting the clinical relevance of in vitro findings [16].

The biological response to titanium degradation products was a recurring theme in the reviewed literature. In vitro studies demonstrated that titanium particles induced dose-dependent cytotoxicity and oxidative stress in gingival fibroblasts, epithelial cells, macrophages, and osteoblast-like cells [17]. Macrophage exposure to titanium particles resulted in activation of pro-inflammatory signaling pathways and increased secretion of cytokines such as interleukin-1β, interleukin-6, and tumor necrosis factor-α [18]. These inflammatory mediators were shown to influence osteoclast differentiation and activity, suggesting a mechanistic link between titanium particle release and peri-implant bone resorption.

Animal and clinical studies provided further evidence supporting this association. Histological analyses of peri-implant tissues from sites affected by peri-implantitis frequently revealed inflammatory infiltrates containing metallic particles, along with elevated expression of pro-inflammatory cytokines and markers of osteoclastic activity [19]. Although causality could not be definitively established, the spatial and temporal association between titanium debris accumulation, inflammation, and bone loss suggested a contributory role of bio-tribocorrosion in peri-implant disease progression [20].

Collectively, the results of the reviewed studies indicate that bio-tribocorrosion is a multifactorial process driven by the interplay of mechanical loading, electrochemical conditions, surface characteristics, and biological activity. The release of titanium ions and particles represents a biologically relevant outcome of this process, with potential implications for peri-implant tissue health and implant longevity. However, significant variability in experimental models and outcome measures was noted across studies, underscoring the need for standardized testing protocols and clinically relevant bio-tribocorrosion models [21]. To clarify the strength and nature of the available evidence and to avoid causal overinterpretation, an evidence-level summary of the included literature is provided in Table 1.

**Table 1:** Evidence-level summary of bio-tribocorrosion findings related to titanium dental implants Table credits: Dr. Shankar S Menon

Evidence Domain	Study Types Included	Key Findings	Strengths	Key Limitations	Level of Inference
Mechanistic (experimental)	In vitro tribocorrosion models; cell culture studies	Mechanical wear disrupts the TiO₂ passive layer, leading to increased corrosion currents and titanium ion/particle release; titanium particles induce dose-dependent oxidative stress and pro-inflammatory cytokine expression in macrophages, fibroblasts, and osteoblast-like cells [3,4,6,8,17,18]	High experimental control; reproducible mechanistic insights; clear dose–response relationships	Loading conditions and exposure concentrations may exceed clinical reality; simplified biological environments	Strong mechanistic plausibility
Preclinical (animal)	Animal implantation and exposure models	Local inflammatory reactions and bone remodeling changes observed in the presence of metallic wear debris, supporting biological reactivity of titanium degradation products [19,20]	Incorporates host immune response and tissue-level effects	Species differences; limited simulation of oral loading and biofilm conditions	Supportive, non-causal
Retrieval/observational	Implant retrieval analyses; peri-implant tissue histology and spectroscopy	Titanium particles and corrosion features detected in peri-implant soft tissues and bone adjacent to failed or diseased implants [8,15,16,22]	Direct assessment of clinically used implants and surrounding tissues	Cross-sectional design; selection bias toward failed implants	Associative
Clinical (human)	Cross-sectional and case-based studies	Elevated titanium ion concentrations and metallic debris associated with peri-implant inflammation and bone loss in peri-implantitis sites [16,20]	High clinical relevance; real-world exposure	No temporal sequence; confounding factors; causality cannot be inferred	Associative, hypothesis-generating
Systemic distribution	Primarily experimental and preclinical studies; limited human observations	Titanium particles detected beyond peri-implant tissues, indicating biological distribution potential rather than established systemic risk [11,17]	Demonstrates transport and dissemination capability	Clinical significance and health impact remain uncertain	Exploratory

To enhance clarity and facilitate comparison of material-specific findings, Table 2 summarizes the tribocorrosion behavior of commercially pure titanium (CpTi) and Ti-6Al-4V titanium alloy used in dental implants, as reported in the reviewed literature.

**Table 2:** Comparative tribocorrosion behavior of commercially pure titanium (CpTi) and Ti-6Al-4V used in dental implants under oral conditions CpTi, commercially pure titanium; TiO₂, titanium dioxide; Al, aluminum; V, vanadium. Table credits: Dr. Shankar S Menon

Parameter	Commercially Pure Titanium (CpTi)	Ti-6Al-4V Alloy
Chemical composition	≥99% titanium with trace impurities	Titanium alloyed with aluminum (6%) and vanadium (4%)
Mechanical properties	Moderate strength; lower hardness compared to alloys	Higher strength and hardness; improved fatigue resistance
Passive oxide layer	Stable TiO₂ layer with good corrosion resistance	Mixed oxide layer (TiO₂ with Al₂O₃ and V oxides)
Wear resistance	Lower wear resistance under high mechanical load	Higher wear resistance due to increased hardness
Corrosion resistance at neutral pH	Excellent corrosion resistance in saliva-like environments	Comparable or slightly reduced resistance depending on microstructure
Behavior under acidic conditions	Increased corrosion rate with impaired repassivation	Similar susceptibility; alloying elements may influence oxide stability
Tribocorrosion susceptibility	Significant material loss under combined wear and corrosion	Reduced wear but complex electrochemical behavior during tribocorrosion
Repassivation kinetics	Rapid repassivation, but repeated mechanical disruption increases cumulative damage	Repassivation occurs but may be altered by alloying oxides
Response to fluoride exposure	Fluoride ions destabilize TiO₂ layer, especially at low pH	Fluoride exposure may promote selective dissolution and oxide instability
Titanium particle release	Release of Ti ions and particles under bio-tribocorrosive conditions	Release of Ti along with potential Al and V ions
Biological response to degradation products	Induces inflammatory cytokine release and macrophage activation	Similar inflammatory response with additional concern for Al/V ion bioreactivity
Association with peri-implantitis	Titanium particles detected in peri-implant tissues in diseased sites	Metallic debris also detected; clinical significance under investigation
Clinical usage trend	Widely used for implant bodies	Commonly used for abutments and some implant systems
Overall tribocorrosion performance	Biocompatible but susceptible to cumulative damage	Mechanically robust but electrochemically complex

Discussion

The clinical evidence referenced in this review primarily originates from university-based academic dental hospitals and tertiary care centers, where implant placement, retrieval analysis, and peri-implant tissue sampling are more commonly performed under controlled research protocols [15,16]. These settings typically involve complex or advanced cases, including peri-implantitis management and implant retrieval, which may overrepresent severe disease presentations. In contrast, data from private practice cohorts remain limited and are often indirect, derived from clinical outcomes rather than material or histological analyses. This imbalance highlights the need for broader clinical investigations encompassing routine private practice populations to better reflect real-world implant exposure, maintenance variability, and long-term material behavior.

Bio-tribocorrosion as a Multifactorial Degradation Process

The findings synthesized in this review highlight bio-tribocorrosion as a complex, multifactorial degradation phenomenon that plays a potentially significant yet underrecognized role in the long-term performance of dental implants. Unlike conventional corrosion or wear processes considered in isolation, bio-tribocorrosion reflects the dynamic and synergistic interaction between mechanical loading, electrochemical reactions, and biological activity within the oral environment. This interaction results in material degradation patterns that are qualitatively and quantitatively distinct from those observed under static or simplified experimental conditions [6].

A central mechanistic feature of tribocorrosion in titanium dental implants is the repeated disruption and reformation of the passive titanium dioxide layer. Titanium relies heavily on this oxide film for its corrosion resistance and biocompatibility; however, mechanical actions such as fretting, micromovement, and abrasion compromise its integrity [7]. While repassivation is generally rapid, repeated mechanical challenges under functional loading conditions prevent complete stabilization of the oxide layer, leading to cumulative material loss over time [8]. This cyclic depassivation-repassivation process is particularly relevant in mechanically stressed regions such as the implant-abutment interface, where microgaps and relative motion may persist to some degree despite advances in implant design [14].

Mechanical and Material Determinants of Bio-tribocorrosion

The implant-abutment interface has emerged as a critical site for bio-tribocorrosion due to the convergence of mechanical, chemical, and biological stressors. Fretting corrosion at this junction is exacerbated by cyclic occlusal forces, manufacturing tolerances, and differences in material composition between implant and abutment components [10]. Electrochemical coupling between dissimilar metals can further enhance corrosion through galvanic interactions, particularly in the presence of saliva acting as an electrolyte [11]. These localized degradation processes not only compromise the structural integrity of the implant system but also facilitate the release of metallic debris into adjacent peri-implant tissues [15].

Surface topography represents another important determinant of tribocorrosion behavior. While moderately rough surfaces are widely recognized for their superior osseointegration potential, increased surface roughness also enhances susceptibility to mechanical wear and localized corrosion by increasing surface area and stress concentration at asperities [9]. The present synthesis suggests that surface modifications optimized solely for bone integration may inadvertently predispose implants to accelerated bio-tribocorrosion under functional loading. This trade-off underscores the need for a balanced approach to surface engineering that accounts for both biological integration and long-term material stability [27].

Chemical and Biological Modifiers in the Oral Environment

The oral environment introduces chemical challenges that further modulate tribocorrosion processes. Fluctuations in pH resulting from dietary intake, microbial metabolism, and inflammatory conditions can significantly influence corrosion kinetics [11]. Acidic environments reduce the stability of the titanium oxide layer and slow repassivation, thereby increasing susceptibility to material degradation following mechanical damage. The presence of fluoride ion compounds this effect by forming soluble complexes with titanium, particularly under low pH conditions, leading to enhanced dissolution of the oxide layer [12]. These findings are clinically relevant given the widespread use of fluoride-containing oral hygiene products and professional prophylactic agents in implant patients [25].

Biological factors distinguish bio-tribocorrosion from purely physicochemical degradation mechanisms. Oral biofilms alter local electrochemical conditions through oxygen depletion, acid production, and accumulation of corrosive metabolites [13]. The formation of biofilms within protected niches such as the implant-abutment microgap promotes localized corrosion phenomena, including crevice corrosion, which may persist even in the absence of overt mechanical wear [22]. Furthermore, biofilm components may interfere with oxide repassivation processes, prolonging exposure of the titanium substrate to corrosive conditions after mechanical disruption [23].

Titanium Degradation Products and Biological Responses

The release of titanium ions and particles represents a biologically significant consequence of bio-tribocorrosion. Evidence from both experimental and clinical studies indicates that these degradation products are not confined to the implant surface but can accumulate in peri-implant soft tissues and bone [15,16]. Particle size appears to be a critical determinant of biological response, with smaller particles, particularly nanoparticles, exhibiting greater reactivity and potential for cellular internalization [17]. This observation aligns with findings from orthopedic implant research, where metallic wear debris has been implicated in inflammatory osteolysis and implant loosening [19].

At the cellular level, titanium degradation products have been shown to induce oxidative stress, membrane damage, and inflammatory signaling in multiple cell types relevant to peri-implant tissue health [18]. Macrophages play a central role in mediating these responses, as phagocytosis of titanium particles triggers the release of pro-inflammatory cytokines that can influence osteoclast differentiation and activity [19]. The resulting imbalance between bone formation and resorption provides a plausible mechanistic link between bio-tribocorrosion and peri-implant bone loss [20].

Clinical Implications

Clinical observations further support this association, as peri-implant tissues affected by inflammatory disease frequently show the presence of metallic particles and elevated inflammatory markers [16]. Although the directionality of this relationship remains difficult to establish, it is increasingly evident that inflammation and bio-tribocorrosion may act in a bidirectional manner. Inflammatory conditions characterized by acidic pH and increased oxidative stress may accelerate material degradation, while degradation products, in turn, exacerbate local inflammation and tissue breakdown [21].

Despite growing evidence implicating bio-tribocorrosion in peri-implant pathology, it remains insufficiently integrated into routine clinical decision-making. Current diagnostic frameworks for peri-implant disease emphasize microbial factors and host immune responses, with limited consideration of implant material degradation as a contributory variable [28]. This conceptual gap may partly explain inconsistencies in treatment outcomes and the variable progression of peri-implantitis observed among patients with similar microbial and systemic risk profiles.

The heterogeneity of experimental models used to study tribocorrosion presents a significant challenge in translating findings to clinical practice. Many in vitro studies employ simplified loading conditions, static electrolytes, and sterile environments that fail to replicate the dynamic mechanical forces, biofilm interactions, and inflammatory milieu of the oral cavity [29]. While such models are valuable for isolating specific mechanisms, they may underestimate the complexity and magnitude of bio-tribocorrosion occurring in vivo. Consequently, there is a need for standardized testing protocols that incorporate mechanical loading, electrochemical monitoring, and biological components to more accurately simulate clinical conditions [21].

Preventive, Therapeutic, and Research Perspectives

From a clinical perspective, the recognition of bio-tribocorrosion necessitates a broader understanding of implant success and failure beyond traditional parameters of osseointegration and plaque control. While microbial biofilms remain a central etiological factor in peri-implant disease, the present synthesis suggests that implant material degradation may act as a parallel and interacting pathway contributing to peri-implant tissue breakdown [20]. Apart from multifactorial contributors such as persistent biofilm, host susceptibility, and access limitations, this perspective may help explain why peri-implantitis can progress despite adequate mechanical debridement and antimicrobial therapy in certain cases, as the underlying material-related stimulus may persist even after microbial load reduction [28].

The bidirectional relationship between inflammation and bio-tribocorrosion warrants particular attention. Inflammatory processes associated with peri-implant mucositis and peri-implantitis create local environments characterized by acidic pH, increased enzymatic activity, and elevated levels of reactive oxygen species, all of which are known to destabilize the titanium oxide layer [11,21]. These conditions may accelerate corrosion and facilitate the release of additional titanium ions and particles, thereby perpetuating inflammation and bone resorption. Such a self-reinforcing cycle underscores the importance of early intervention and strict maintenance protocols to prevent the establishment of chronic inflammatory conditions around dental implants [25].

Prosthetic design and occlusal considerations also play a significant role in modulating bio-tribocorrosion. Excessive occlusal loading, parafunctional habits, and unfavorable force distribution increase micromovements at the implant-abutment interface, thereby enhancing fretting corrosion and wear [14]. Platform switching, improved connection geometries, and optimized torque application have been proposed as strategies to reduce micromotion and minimize mechanical disruption of the oxide layer [10]. However, while these design modifications may mitigate mechanical wear, their long-term effectiveness in reducing bio-tribocorrosion under complex oral conditions remains incompletely understood.

Material selection represents another critical factor influencing tribocorrosion behavior. Although commercially pure titanium and titanium alloys have demonstrated acceptable clinical performance, differences in alloy composition and microstructure influence wear resistance, corrosion kinetics, and oxide stability [26]. Ti-6Al-4V, while mechanically robust, has raised concerns regarding the release of aluminum and vanadium ions, which may exert additional biological effects when released in conjunction with titanium particles [18]. Though these findings are primarily experimental or in vitro, with uncertain clinical significance, these considerations have prompted investigation into alternative titanium alloys and novel biomaterials designed to enhance corrosion resistance while maintaining favorable mechanical properties [27].

Surface modification strategies aimed at improving osseointegration may also influence susceptibility to bio-tribocorrosion. Techniques such as sandblasting, acid etching, anodization, and plasma spraying create micro- and nanoscale surface features that enhance bone-implant contact but may simultaneously increase surface reactivity and vulnerability to mechanical wear [9]. Recent advances in surface coatings, including ceramic layers, diamond-like carbon, and bioactive coatings, have shown promise in reducing wear and corrosion while preserving biological compatibility [27]. Nevertheless, the durability of such coatings under long-term functional loading and biofilm exposure requires further investigation before widespread clinical adoption.

Preventive strategies targeting bio-tribocorrosion must be multifaceted, addressing mechanical, chemical, and biological contributors simultaneously. From a clinical standpoint, careful prosthetic planning to minimize occlusal overload, regular maintenance to control biofilm accumulation, and cautious use of acidic or highly concentrated fluoride agents in implant patients are prudent measures supported by current evidence [12,25]. Patient-specific risk factors, including parafunctional habits, systemic inflammatory conditions, and oral hygiene practices, should also be considered when developing long-term maintenance protocols [28].

The therapeutic implications of titanium particle release are an emerging area of interest. While mechanical debridement and surgical intervention remain the mainstay of peri-implantitis management, the persistence of metallic debris within peri-implant tissues may limit treatment efficacy [16]. Some authors have suggested that implant surface detoxification and decontamination strategies should account not only for microbial biofilms but also for the presence of corrosion products and wear debris [20]. However, standardized clinical protocols addressing this aspect are currently lacking, reflecting a broader gap between experimental findings and clinical practice.

From a research perspective, the findings synthesized in this review highlight significant gaps in the current understanding of bio-tribocorrosion. Standardized experimental models that incorporate cyclic mechanical loading, electrochemical monitoring, and biologically relevant conditions are urgently needed to improve comparability across studies [21,29]. In vivo investigations and well-designed clinical studies correlating titanium degradation products with peri-implant disease progression would further strengthen causal inference and translational relevance [16].

Additionally, the long-term biological fate of titanium particles released through bio-tribocorrosion remains incompletely understood. While local inflammatory effects have been demonstrated, the potential for systemic distribution and distant biological effects warrants further investigation, particularly in patients with multiple implants or compromised immune regulation [15,17]. Advances in analytical techniques may facilitate more precise characterization of particle size, composition, and biological interactions, thereby enhancing understanding of their clinical significance.

Finally, the concept of bio-tribocorrosion challenges clinicians and researchers to adopt a more integrative view of implant dentistry, recognizing that implant success is governed by the interplay between material science, biomechanics, microbiology, and host biology [30]. Incorporating this paradigm into implant design, clinical protocols, and maintenance strategies may ultimately contribute to improved long-term outcomes and reduced incidence of peri-implant complications.

Study Limitations and Future Directions

Several limitations inherent to the narrative review design must be acknowledged. First, the absence of standardized tribocorrosion testing protocols across studies limits direct comparison of outcomes and hinders quantitative synthesis. Experimental conditions vary widely with respect to mechanical loading regimes, electrochemical environments, surface characteristics, and biological models, introducing methodological heterogeneity. Second, a substantial proportion of the available evidence is derived from in vitro investigations conducted under simplified and often sterile conditions that do not fully replicate the dynamic mechanical forces, complex biofilm ecosystems, and host immune responses present in the oral cavity. Consequently, extrapolation of experimental findings to clinical scenarios should be undertaken with caution [15,17].

Future private-practice studies should document maintenance protocols, fluoride exposure patterns, prosthetic designs, and occlusal loading to enhance external validity and translational relevance [15,16]. Future research should prioritize the development of standardized, biologically relevant tribocorrosion models incorporating cyclic mechanical loading, electrochemical monitoring, and microbial or inflammatory components to better simulate intraoral conditions. Longitudinal clinical and retrieval studies correlating titanium degradation products with peri-implant tissue changes would strengthen causal inference and translational relevance. Additionally, further investigation into advanced surface coatings, alternative alloys, and prosthetic design modifications is warranted to mitigate bio-tribocorrosion and improve long-term implant performance [30].

## Conclusions

Bio-tribocorrosion of titanium dental implants is a multifactorial phenomenon influenced by mechanical loading, electrochemical interactions, oral biofilm activity, and patient-related factors such as saliva composition and parafunctional habits. The available literature suggests that the synergistic interaction between wear and corrosion can compromise implant surface integrity, promote titanium particle release, and potentially contribute to peri-implant tissue inflammation and long-term implant complications. However, the extent to which bio-tribocorrosion directly impacts clinical implant failure remains incompletely understood.

Future research should focus on standardized in vitro and in vivo models that better simulate the complex oral environment, as well as long-term clinical studies correlating tribocorrosion-related surface changes with biological and clinical outcomes. A clearer understanding of these mechanisms will aid in the development of improved implant materials, surface modifications, and preventive strategies aimed at enhancing implant longevity and peri-implant health.

## Appendices

The electronic search strategy, including databases searched, Boolean operators, and MeSH or controlled vocabulary terms, is summarized in Table 3. The search timeframe was from database inception through January 2026. Only articles in English language were considered. Study types included in vitro, animal, implant retrieval, and clinical observational studies relevant to oral implantology.

**Table 3:** Electronic search strategy and MeSH terms used Credits: Dr. Shankar S Menon

Database	Search Strategy (Boolean Combination)	MeSH Terms/Controlled Vocabulary
PubMed/MEDLINE	(“tribocorrosion” OR “bio-tribocorrosion” OR (“wear” AND “corrosion”) OR “fretting corrosion”) AND (“titanium” OR “titanium alloys” OR “Ti-6Al-4V”) AND (“dental implants” OR “implant–abutment interface” OR “peri-implantitis”)	Dental Implants; Titanium; Titanium Alloys; Corrosion; Wear; Peri-Implantitis; Biofilms
Scopus	TITLE-ABS-KEY (tribocorrosion OR bio-tribocorrosion OR wear-corrosion OR fretting) AND (titanium OR titanium alloy) AND (dental implant* OR peri-implantitis OR implant-abutment)	Indexed keywords (database-specific; no MeSH)
Web of Science	TS = (tribocorrosion OR bio-tribocorrosion OR fretting corrosion) AND TS = (titanium AND dental implants)	Keywords Plus / Topic terms
Manual search	Reference list screening of key reviews and highly cited primary articles	Not applicable
